# Is perfect prompting possible for chatbots? In Reply to: Comparing ChatGPT’s ability between writing versus reviewing papers – then what?

**DOI:** 10.3325/cmj.2024.65.399

**Published:** 2024-08

**Authors:** Gültekin Kadi, Mehmet Ali Aslaner

**Affiliations:** Department of Emergency Medicine, Gazi University Faculty of Medicine, Ankara, Turkey; *gultekinkadi@gazi.edu.tr*

We are grateful to Dr Matsubara for his comments and have carefully considered the listed remarks. Although the author's concerns are justified, we would like to clarify some points.

First, our study question was whether AI can independently create a case report with an original title and other inputs used as prompts. Dr Matsubara is right about the importance of using specific input when evaluating ChatGPT's capabilities, and about entering a detailed prompt for a more specific output. In future experiments, different inputs may be tested. As can be seen from case studies in the literature, there has yet to be a perfect or standard prompt, and it remains unclear which prompt gives better output (1).

Second, the case reports generated by ChatGPT in our pilot studies contained made-up references. This happened even though we gave specific instructions to “Include in-text citations where appropriate for at least three references from real published original articles.” In other studies as well, AI created non-existent and irrelevant references (2). On the other hand, when in our study ChatGPT peer-reviewed a case report without a reference section, it rated the reference section as good. The report only scored poor in terms of writing quality. AI was not able to establish a relationship between manuscript sections (3).

Third, prompt engineering, a new concept, is developing faster every day (4). It is possible to develop personalized prompts for different research purposes and jobs. Just as the same output can be obtained with different prompts, different outputs can also be achieved with the same prompts. We want to highlight that AI is both a threat and an opportunity for academic publishing. We ethically and personally believe that AI can be used just to make writing easier, not to produce content.

Finally, we generated two outputs based on two different inputs to produce a case report: one is Kadi and Aslaner's prompt and the other is Matsubara's prompt (Supplemental Material 1(Supplementary Material)). Readers, decide!
