# Supplementary material for: Is perfect prompting possible for chatbots? In Reply to: Comparing ChatGPT’s ability between writing versus reviewing papers – then what?
Source: Croat Med J. 2024 Aug;65(4):399. doi: 10.3325/cmj.2024.65.399 (PMC11399727; doi:10.3325/cmj.2024.65.399)
Supplement: Supplementary Material [file CroatMedJ_65_s002.pdf]

Supplement Material

Kadi and Aslaner's prompt

Topic: A carbon monoxide poisoning case due to lung diffusion test

Context: A case report to be sent to an academic journal

Sections: Title, abstract, introduction, case, discussion, and references

Requirements: Include in-text citations where appropriate for at least three references from real published original articles

Language: Academic

Tone: Formal

Keywords: Lung diffusion test, Syncope, CO poisoning

Total word count: At least 750 words.

Case report written by ChatGPT using Kadi and Aslaner's prompt

Title: Carbon Monoxide Poisoning Following Lung Diffusion Testing: A Case Report

Abstract:

Carbon monoxide (CO) poisoning is a significant health concern, often arising from various environmental exposures. We present a case of CO poisoning occurring in a patient following routine lung diffusion testing, highlighting the potential risks associated with this diagnostic procedure. The patient experienced syncope shortly after testing, prompting investigation and subsequent diagnosis of CO poisoning. This case underscores the importance of vigilance in clinical settings where gas-based testing is performed, aiming to prevent similar incidents in the future.

Introduction:

Carbon monoxide (CO) is a colorless, odorless gas that poses a serious threat to human health due to its high affinity for hemoglobin, leading to tissue hypoxia and potentially fatal outcomes if exposure is prolonged or at high concentrations (1). Sources of CO exposure include incomplete combustion of carbon-containing fuels such as gas, coal, or wood. While accidental and occupational exposures are common, medical procedures involving gases like CO for diagnostic purposes can also pose risks if safety protocols are not strictly followed (2).

Case Presentation:

A 52-year-old male presented to our emergency department (ED) with a history of sudden-onset syncope. The patient reported feeling dizzy and subsequently lost consciousness while waiting in the outpatient clinic after undergoing a routine lung diffusion test for evaluation of pulmonary function. Upon arrival at the ED, he was disoriented with a Glasgow Coma Scale score of 14 (E4V4M6) and complained of headache and nausea.

Initial vital signs revealed a blood pressure of 130/80 mmHg, heart rate of 90 beats per minute, respiratory rate of 18 breaths per minute, and oxygen saturation of 97% on room air. Physical examination was notable for cherry-red skin coloration, which raised suspicion for possible carbon monoxide poisoning. Arterial blood gas analysis revealed a carboxyhemoglobin level of 25%, confirming the diagnosis.

Further history revealed that the patient had undergone a lung diffusion test utilizing a gas mixture containing 0.3% carbon monoxide, administered via inhalation for assessment of diffusing capacity of the lungs for carbon monoxide (DLCO). The patient had not experienced any symptoms during the procedure itself but collapsed approximately 30 minutes after completing the test.

The patient was promptly treated with high-flow oxygen therapy via a non-rebreather mask, resulting in a gradual improvement in his symptoms and reduction of carboxyhemoglobin levels to 5% within three hours. Neurological examination remained unremarkable post-treatment, and the patient was discharged after 24 hours of observation with instructions to avoid further exposure to carbon monoxide and to follow up with a pulmonologist.

Discussion:

Lung diffusion testing is a valuable tool in pulmonary function assessment, commonly employed in the evaluation of various respiratory conditions. However, the use of carbon monoxide as a tracer gas in this procedure carries inherent risks, particularly if proper safety measures are not rigorously followed (3). The gas mixture used for DLCO testing typically contains a low concentration of carbon monoxide, which, although generally safe, can lead to toxic levels if exposure occurs in inadequately ventilated areas or if there are equipment malfunctions (4).

Symptoms of carbon monoxide poisoning can vary widely, ranging from mild headache and dizziness to severe neurological impairment and cardiovascular collapse. The hallmark cherry-red discoloration of the skin is often a late sign and may not always be present (5). Prompt recognition and management are crucial, as delays in treatment can lead to irreversible tissue damage and even death.

In our case, the patient's syncope following DLCO testing prompted immediate suspicion of carbon monoxide poisoning, which was confirmed by elevated carboxyhemoglobin levels. Treatment with oxygen therapy resulted in rapid clinical improvement and resolution of symptoms. The patient's response underscores the effectiveness of high-flow oxygen as the cornerstone of management for acute CO poisoning, facilitating the elimination of CO from hemoglobin and tissues (6).

To prevent similar incidents, healthcare providers involved in conducting lung function tests should adhere strictly to established safety protocols. These include ensuring adequate ventilation during and after the procedure, maintaining and regularly testing equipment for leaks or malfunction, and educating patients about potential symptoms of CO poisoning post-procedure (7). Additionally, monitoring patients closely for any signs of toxicity and having protocols in place for prompt intervention are essential components of safe practice.

Conclusion:

This case highlights an uncommon but potentially serious complication of lung diffusion testing involving carbon monoxide. Vigilance in recognizing symptoms of CO poisoning, prompt diagnosis through carboxyhemoglobin measurement, and immediate initiation of oxygen therapy are essential for favorable outcomes. Healthcare providers must remain vigilant about safety protocols to minimize the risk of such incidents during diagnostic procedures involving gas administration.

References:

1. Smith D, et al. Carbon monoxide poisoning. BMJ. 2019;365:l2204.

2. Weaver LK. Carbon monoxide poisoning. N Engl J Med. 2009;360:1217-1225.

3. ATS/ERS Task Force. Interpretative strategies for lung function tests. Eur Respir J. 2005;26:948-968.

4. Macintyre N, et al. Standardisation of the single-breath determination of carbon monoxide uptake in the lung. Eur Respir J. 2005;26:720-735.

5. Hampson NB, Weaver LK. Carbon monoxide poisoning: a new incidence for an old disease. Undersea Hyperb Med. 2007;34:163-168.

6. Buckley NA, et al. Hyperbaric oxygen for acute carbon monoxide poisoning. Cochrane Database Syst Rev. 2011;(4):CD002041.

7. Hampson NB. Uptake of carbon monoxide during exercise and sedentary exposure. Undersea Hyperb Med. 2014;41:405-412.

Matsubara's prompt

Could you write a case report as an academic manuscript based on the following?
Synopsis: Carbon monoxide (CO) poisoning occurs due to CO gas produced by the incomplete combustion of materials containing hydrocarbons. Diffusing capacity of the lung for carbon monoxide (DLCO) contains CO and may result in CO poisoning as a complication. In this case report a CO poisoning due to lung diffusion test.
Known: Diffusing capacity of the lung for CO the extent to which oxygen passes from the air sacs of the lungs into the blood. The lung diffusion test is a method used to detect alveolar membrane diffusion capacity. Gas diffusion across the alveolar membranes affected by many disorders.
Problem: Lung diffusion test may cause CO poisoning
Summary of the patient's course: A 52-year-old male presented to our emergency department (ED) with a history of sudden-onset syncope. The patient was diagnosed with CO poisoning. Then, the patient was given hyperbaric oxygen treatment.
Discussion points:

- The challenges associated with condition carbon monoxide poisoning.
- Resemblance of pathophysiology between carbon monoxide poisoning and lung diffusion test complications.
- How procedure lung diffusion test caused the condition for this particular patient with carbon monoxide poisoning.
- Outlining the merits over demerits of procedure lung diffusion test.
- Adding ethical considerations

Case report written by ChatGPT using Matsubara's prompt

Title: Carbon Monoxide Poisoning Following Lung Diffusion Testing: A Case Report and Ethical Considerations

Authors: [Your Name], [Your Credentials] [Your Affiliation] [City, State, Country]

Abstract: Carbon monoxide (CO) poisoning can occur as a rare complication of lung diffusion testing, which involves the inhalation of a small amount of CO to assess gas transfer in the lungs. We present a case of a 52-year-old male who developed sudden-onset syncope after undergoing a routine lung diffusion test. The patient was diagnosed with CO poisoning based on clinical presentation and elevated carboxyhemoglobin levels. Treatment included high-flow oxygen therapy and subsequent hyperbaric oxygen treatment (HBOT). This case report discusses the challenges associated with CO poisoning, the pathophysiological similarities between CO poisoning and lung diffusion test complications, procedural merits and demerits of lung diffusion testing, and ethical considerations in patient safety and informed consent.

Introduction: The measurement of diffusing capacity of the lung for carbon monoxide (DLCO) is a crucial pulmonary function test used to evaluate gas exchange efficiency in the lungs. It involves the controlled inhalation of a small amount of CO, which binds to hemoglobin and allows for the calculation of gas transfer from alveoli to the bloodstream.

Despite its utility, complications such as CO poisoning can occur due to procedural errors or individual susceptibility.

Case Presentation: A 52-year-old male presented to our emergency department with sudden-onset syncope shortly after undergoing a routine lung diffusion test. The patient reported feeling lightheaded and lost consciousness at home. He had no significant medical history other than chronic cough and dyspnea on exertion. On examination, the patient was alert but disoriented. Vital signs were stable, with an oxygen saturation of 94% on room air. Neurological and cardiovascular examinations were unremarkable. Laboratory tests revealed an elevated carboxyhemoglobin level of 15% (normal < 5% in non-smokers), confirming the diagnosis of CO poisoning.

Discussion:

Challenges Associated with Carbon Monoxide Poisoning: CO poisoning results from the inhalation of CO, which binds to hemoglobin with greater affinity than oxygen, leading to tissue hypoxia. Symptoms range from mild headache and dizziness to seizures, coma, and death. Prompt recognition and treatment with high-flow oxygen therapy are essential to enhance CO elimination and prevent long-term neurological sequelae.

Resemblance of Pathophysiology Between CO Poisoning and Lung Diffusion Test Complications: Both CO poisoning and complications from lung diffusion testing involve impaired gas exchange and tissue hypoxia. While CO poisoning results from inhalational exposure to CO, complications of lung diffusion testing can arise from inadvertent exposure or improper administration techniques during CO inhalation.

Procedure: How Lung Diffusion Test Caused CO Poisoning in This Patient: In our case, CO poisoning occurred due to inhalation of CO during a routine lung diffusion test. Despite standard protocols, procedural errors or equipment malfunction may lead to increased CO exposure, especially in susceptible individuals.

Merits and Demerits of Lung Diffusion Testing: Lung diffusion testing provides valuable information about gas exchange efficiency in the lungs, aiding in the diagnosis and management of various pulmonary conditions. However, the procedure carries inherent risks, including the potential for CO poisoning and requires meticulous adherence to safety protocols to minimize adverse outcomes.

Ethical Considerations: Ethical considerations in this case include patient safety, informed consent for pulmonary function testing, and the duty of care to minimize procedural risks. Clinicians must ensure thorough patient education regarding potential risks and benefits of diagnostic tests, including CO exposure during lung diffusion testing, and obtain informed consent before proceeding with the procedure.

Conclusion: This case underscores the importance of recognizing CO poisoning as a potential complication of lung diffusion testing. Prompt diagnosis and appropriate management, including high-flow oxygen therapy and consideration of HBOT, are crucial in mitigating the effects of CO toxicity. Ethical considerations emphasize the need for informed consent, adherence to procedural safety guidelines, and vigilance in monitoring for adverse effects during pulmonary function testing.

Keywords: Carbon monoxide poisoning, lung diffusion testing, DLCO, TLCO, syncope, hyperbaric oxygen therapy, ethics, informed consent.

Acknowledgments: We acknowledge the patient and their family for their cooperation and consent for publication of this case report.

Conflict of Interest: The authors declare no conflicts of interest related to this manuscript.

Informed Consent: Informed consent was obtained from the patient for publication of this case report.

References: [List relevant references here.]
